# Digitally-mediated coordination in healthcare: the effects of teleconsultation on doctor-to-doctor relational coordination

**DOI:** 10.1186/s12913-024-10726-5

**Published:** 2024-02-28

**Authors:** Mattia Vincenzo Olive, Luca Gastaldi, Mariano Corso

**Affiliations:** Department of Management, Economics and Industrial Engineering, Politecnico di Milano, Milan, Italy

**Keywords:** Relational coordination, Coordination, Organization, Telemedicine, Healthcare

## Abstract

**Background:**

Digitalization transforms the way in which interdependent work is coordinated, especially in healthcare settings. This work deepens the effect of teleconsultation use on health professionals’ coordination. For this aim, we rely on Relational Coordination Theory (RCT), which explores coordination as an interactive process among group participants within the context of task interdependency.

**Methods:**

We collected data through an online survey administered to Italian specialist doctors between March and April 2023. 489 complete answers were gathered. Hypotheses have been tested through Structural Equation Modelling.

**Results:**

We found that teleconsultation frequency of use has a positive and significant effect on both components of relational coordination, confirming our hypotheses.

**Conclusions:**

Theoretically, this research contributes to our understanding of the effect of digitally mediated coordination mechanisms on relational coordination. In practice, we shed light on the organizational implications of telemedicine under a novel perspective, focusing on the role of professional interactions in digitally mediated work and providing useful elements for the organizational design of telemedicine.

**Supplementary Information:**

The online version contains supplementary material available at 10.1186/s12913-024-10726-5.

## Background

The growing complexity of patients’ needs [1, 2] requires the effective coordination [1, 3–5] of ‘hyper-specialized’ healthcare workforce [6]. Digitalization can play a pivotal role in this challenge [4, 7], transforming the way in which interdependent knowledge work – such as the medical one – is coordinated [8–11]. In particular, teleconsultation ‘digitally mediates’ coordination [12], affecting the nature of communications and, therefore, the quality of relationships among professionals [3, 13, 14]. This latter aspect is particularly relevant since it has been demonstrated that high-quality communication and information sharing in coordination processes lead to better health outcomes [4, 15–17].

Starting from these premises, in this study we leverage on Relational Coordination Theory (RCT) [18] and ask: *does the use of teleconsultation among physicians strengthen relational coordination?* To answer this question, we performed statistical analyses on data gathered through a survey in Italy, testing our hypotheses on *specialist doctors* interacting among themselves and with *General Practitioners (GPs),* within the empirical context of multidisciplinary, multi-setting care.

Theoretically, we contribute to understanding the impacts of digitally-mediated coordination mechanisms [8] on relational coordination [19]. While previous research mainly deepened relational coordination as a key antecedent of organizational outcomes [20–22], very few studies focused on linking it to coordination mechanisms [19]. This gap widens further with respect to digital technologies in general [8] and telemedicine in particular [23].

In practice, we shed light on the organizational implications of teleconsultation from a novel perspective, focusing on the roles of professionals and their digital interactions and providing useful elements for the organizational design of telemedicine services.

### Teleconsultation

Telemedicine influences the organization of medical work, enhancing coordinated care and multidisciplinarity [10, 11, 24–26]. Among telemedicine applications, doctor-to-doctor teleconsultation is peculiarly relevant for medical work [27]. Teleconsultation is the “synchronous or asynchronous consultation using information and communication technology to omit geographical and functional distance” [12] among health professionals. It facilitates communication and data sharing among those professionals who share responsibilities over same patients (e.g., specialist doctors and GPs), as in the management of chronic diseases [28]. It also enables seeking professional advice from medical peers who possess specific knowledge in a particular specialization or sub-specialization [12].

Teleconsultation is acknowledged to enhance efficiency, for instance by saving traveling and waiting times [29, 30]. Moreover, by diminishing the impact of physical distance, it has the potential to reduce geographical barriers, thereby facilitating cooperation and coordination [31].

Surprisingly, although the enabling technologies for teleconsultation are widely available in Western economies [32], these services are still not much widespread [33]. The causes of this late diffusion are variegated in nature and comprise complex implementation processes [33], which typically characterize bureaucratic professional organizations [34, 35].

Within the context of coordinated care, teleconsultation transforms the nature of communications and, therefore, affects the quality of relationships among the involved professionals [3, 13, 14]. Although previous studies discuss how teleconsultation impacts professional dynamics [10] and coordination processes [27, 36], to the best of authors’ knowledge there are no previous studies examining whether and how teleconsultation affects the *quality of relationships* within coordination processes, which was proven to be a relevant explanatory factor for care quality [21, 37].

### Relational Coordination Theory

The concept of *coordination* refers to the process through which organizational agents manage interdependencies and integrate their efforts [38–41]. In very simple terms, coordination answers the question: “how do people work *together* within organizations?”. *Coordination mechanisms* are “the organizational arrangements that allow individuals to realize a collective performance” [40].

Relational Coordination Theory (RCT) argues that coordination mechanisms are “arguably the central elements of what effective groups do” [37], providing a perspective on coordination that refers to the interactions among participants rather than the mechanisms for supporting or replacing these interactions [37].

RCT proposes that “relationships characterized by shared goals, shared knowledge, and mutual respect tend to support frequent, timely, accurate, problem solving communication and vice versa, enabling stakeholders to effectively coordinate their work” [18]. The theory claims that personal relationships are not enough to ensure optimal organizational outcomes, as they are typically difficult to intentionally manage and individual-dependent [8]. Instead, relational coordination unfolds among functional *roles* independently from the specific individuals that represent them (e.g., between physicians and nurses, or physicians and technicians) [42].

Relational coordination is the core concept of this theory and it may be defined as a “mutually reinforcing process of communicating and relating for the purpose of task integration” [19]. Relational coordination encapsulates four dimensions related to *communication* (frequent, timely, accurate, problem-solving-focused) and three related to *relationships* (shared goals, shared knowledge, and mutual respect).

In its original formulation, relational coordination has a ‘mediating’ effect between cross-cutting (in other terms, transversal) organizational structures (such as coordination mechanisms, HR practices for teamwork, etc.) and organizational outcomes of coordination processes [19, 43]. In other terms, RCT proposes that the effect of coordination mechanisms on performance is mediated by the dimensions of relational coordination.

Several works demonstrated the positive effect of relational coordination on coordination outcomes, especially in the healthcare domain [21, 44–47]. However, there is less evidence on the effect of different types of coordination mechanisms on relational coordination [8, 19]. Whereas some findings demonstrate the effect of in-person meetings and interactions in healthcare settings [48–51], physical presence may not be a necessary condition of strong relational coordination [52].

Moreoer, although past organizational studies [8–11, 53, 54] widely recognized that digitalization transforms coordination mechanisms, findings on the impact of digital technologies on relational coordination are scarce and mixed [19]. Claggett & Karahanna [8] not only argued that digitalization may strengthen the ‘structure’ of coordination mechanisms (i.e., increasing the standardization of coordination activities) but also suggested that it is possible to ‘switch’ interactions from a physical to a digital environment, without compromising their effectiveness. This article aims to test this latter aspect.

## Methods

### Hypotheses and models

We developed our hypotheses grounded on RCT [21], in particular on the fact that, on the one hand, coordination mechanisms can support (or be detrimental to) relational coordination [19], while, on the other hand, teleconsultation digitally mediates interactions within coordination processes [8]. On these bases, we advance the following hypotheses:

- *H1a: The frequency of use of teleconsultation between specialist doctors and GPs positively affects the ‘communication’ dimension of relational coordination.*
- *H1b: The frequency of use of teleconsultation between specialist doctors and GPs positively affects the ‘relation’ dimension of relational coordination.*
- *H2a: The frequency of use of teleconsultation among specialist doctors positively affects the ‘communication’ dimension of relational coordination.*
- *H2b: The frequency of use of teleconsultation among specialist doctors positively affects the ‘relation’ dimension of relational coordination.*

**Fig. 1:**
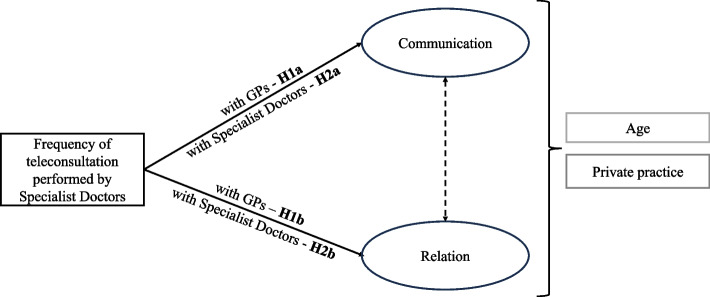
Theoretical model and hypotheses

As specified in Fig. 1, two separate (but specular, in terms of measured constructs) models were set to test the impact of teleconsultation utilization *among specialist doctors* and *with GPs* on relational coordination. The models consider the hypotheses previously stated, as well as the assumption of error covariance between the two dimensions of relational coordination – based on the core proposition of RCT.

### Measures

To measure relational coordination we exploited the ‘Relational Coordination Survey’ [18, 55], which consists of a 7-item scale measuring the dimensions of relational coordination (frequent communication, timely communication, accurate communication, problem-solving communication, shared knowledge, mutual respect, shared goals) through a 1–5 Likert scale. Moreover, it contained *two* measures of relational coordination: one related to specialist doctors (respondents to the survey) and GPs (Cronbach’s α = 0.918), and the other related to relational coordination among specialist doctors (Cronbach’α = 0.897).

Concerning survey adaptation and translation, the items were professionally translated from English to Italian. Following this, in order to ensure accuracy and avoid any potential misinterpretations, a reverse translation was conducted by a different professional translator from Italian back to English. Subsequently, a bilingual practitioner, proficient in both Italian and English, conducted a thorough review of the survey to verify the consistency and accuracy of the translation.

With respect to the original formulation, our survey was not site-specific but it was submitted to a large sample of Italian doctors. Therefore, at the beginning of the relational coordination section, a scenario was given to respondents, which explicitly asked them to envision a specific case in which the two health professionals are in different ‘settings’. In the Italian context, this is inherently true in the case of GPs, who typically work in different organization or as ‘autonomous’ collaborators of a local health authority. The scenario in which the two professionals work for the same institution does not affect the validity of the answer, as the emphasis is put on working in different locations or setting, where distance communication is required.

The frequency of use of teleconsultation was self-reported through a 4-item Likert scale (from ‘Never’ to ‘On a daily basis’), with reference to the precedent year. We asked separately for this information referring to teleconsultations with GPs and with other specialist doctors.

Measured control variables are age and a binary variable to indicate whether the doctor works in private entity (as it could potentially imply a more ‘autonomous’ working habit).^1^

In Additional file 1, the details of the items investigated through the survey are provided.

### Data collection

The survey was administered through email to Italian specialist doctors between March and April 2023, with the support of three medical associations and a health communication agency and in compliance with the European privacy regulation (GDPR). Completion required about 10 min. The email was sent to a nationwide database of approximately 150,000 doctors, with respect to an overall population of around 202,000 specialist doctors [56]. 489 complete answers were gathered.

Table 1 portrays the age distribution of the sample of respondents. Although the stratification of the targeted sample is not known to researchers, we checked that the distribution of responses was *coherent* with the one of the population – both in terms of age as well as gender. In this sense, OECD data [56] show that 55% of Italian doctors in 2022 were 55 or older and 46% were female.

**Table 1:** Age distribution of the sample

**Age group**	**%**
25–30	1,2%
31–35	2,7%
36–40	9,8%
41–45	7,6%
46–50	11,9%
51–55	13,3%
56–60	19,0%
61–65	19,6%
> 65	14,9%
**Gender**	**%**
Male	55%
Female	45%
**Measure**	**Value**
Min	28
Max	76
Mean	55

Sample: 489 Italian Specialized Doctors

**Fig. 2:**
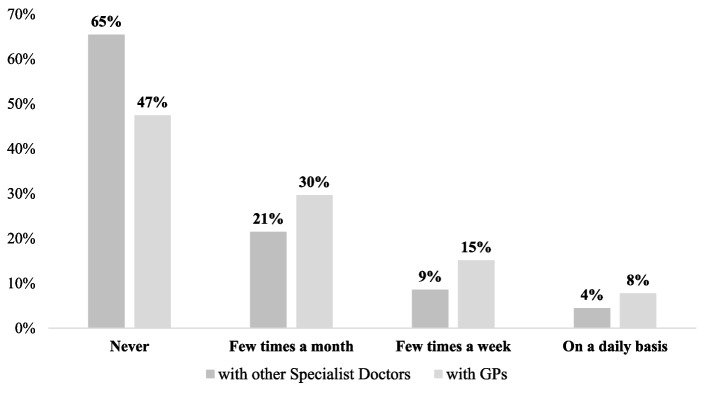
Frequency of teleconsultation performed among specialist doctors and between specialist doctors and GPs. Sample: 489 Italian Specialized Doctors

Figure 2 displays the distribution of respondents according to the frequency with which they performed teleconsultation both with other specialist doctors and with GPs.

### Data analysis

Structural Equation Modeling (SEM) was employed to test our hypotheses, using two separate models to respectively assess the impact of teleconsultation among specialist doctors and with GPs on relational coordination. To control for constructs’ reliability and validity, we calculated the Average Variance Extracted (AVE) and Composite Reliability (CR). As a final step, the goodness of fit of the proposed model was assessed through four indexes, as recommended by literature [57]: the Root Mean Square Error of Approximation (RMSEA), the Standardized Root Mean Residual (SRMR), the Comparative Fit Index (CFI), the Tucker-Lewis Index (TLI). As reported in Table 2, all indexes’ values are in line with common values accepted in the literature [57].

**Table 2:** Good of fit indexes

Indicator	Value	Reference Value
**Specialist-Specialist interaction model**		
RMSEA	0.078	≤ 0.08
SRMR	0.030	≤ 0.08
CFI	0.956	≥ 0.90
TLI	0.935	≥ 0.90
**Specialist-GP interaction model**		
RMSEA	0.088	≤ 0.08
SRMR	0.028	≤ 0.08
CFI	0.955	≥ 0.90
TLI	0.933	≥ 0.90

Analyses were conducted using STATA v14.1.

## Results

### Measurement models

Table 3 displays CR and AVE for the two constructs in the two models. The values are higher than commonly accepted thresholds (0.70 and 0.50), indicating good validity for all constructs [57–59].

**Table 3:** Confirmatory factor analysis

**Latent variable**	**Observed variable**	**Specialist-specialist interaction mode**			**Specialist-GP interaction model**		
		**Factor Loading**	**CR**	**AVE**	**Factor Loading**	**CR**	**AVE**
Communication	Frequent Communication	0.60	0.83	0.56	0.66	0.88	0.66
	Timely Communication	0.79			0.82		
	Accurate Communication	0.81			0.87		
	Problem Solving Communication	0.77			0.88		
Relation	Shared Knowledge	0.83	0.86	0.67	0.82	0.85	0.66
	Mutual Respect	0.81			0.78		
	Shared Goals	0.81			0.83		

### Structural models

**Fig. 3:**
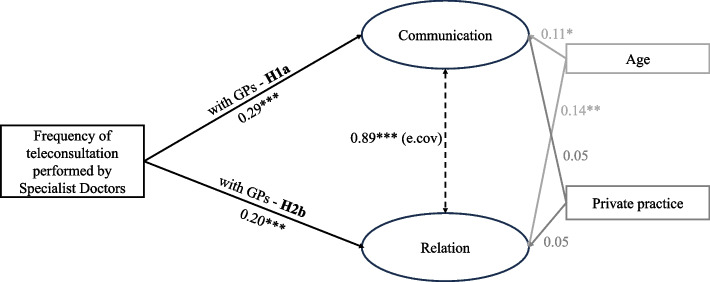
Specialist-GP interaction SEM model: estimates

Figure 3 represents the structural model for the specialist-GP interaction model. Age has a statistically significant and positive effect on both communication and relation while working in private practice seems not to be significant. The path coefficients explaining the effect of the frequency of teleconsultation on both dimensions of relational coordination are statistically significant and positive. Therefore, both H1a and H1b are confirmed.

**Fig. 4:**
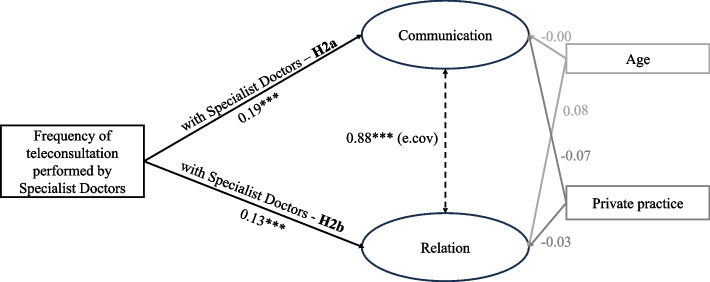
Specialist-specialist interaction SEM model: estimates

Figure 4 represents the structural model for the specialist-specialist interaction model. In this case, none of the control variables appears to significantly affect the dimensions of relational coordination. The path coefficients explaining the effect of the frequency of teleconsultation on both the dimensions of relational coordination (communication and relation) are statistically significant and positive. Therefore, both H2a and H2b are confirmed.

## Discussion

This study deepens the effect of teleconsultation use on health professionals’ coordination, through the lens of RCT. We found that the frequency through which teleconsultation is used by specialist doctors has a positive and significant effect on both the components of relational coordination, confirming our initial hypotheses. In other terms, our evidence shows that the more specialist doctors recur to teleconsultation to interact with both other specialist doctors and GPs, the better their relational coordination – both in terms of communicating and relating.

Based on our findings, we claim that physically distributed work based on expert knowledge [38], such as the case of the medical one, can benefit from the digital medium in coordination processes. The research design put a particular emphasis on chronic patient management, which usually requires the enactment of coordination mechanisms across physical facilities, or even organizations [5, 60]. Therefore, we also argue that spatial proximity is not a necessary condition of strong relational coordination among roles [52], but it consists of one possible design for coordination practices which may be appropriate and beneficial, although not always.

Finally, our findings show a stronger intensity of the two main paths in the specialist-GP model. Although the interpretation of this finding should be careful, in light of the coefficient standardization applied by the statistical model, we can still claim to some extent that the effect of the frequency of use of teleconsultation on relational coordination is stronger in the case of specialist-GP interaction. We argue that these findings are contextual and depend on purely professional dynamics characterizing medical work in terms of jurisdictions, hierarchies, boundary spanners, etc. [34, 61], but indeed this argument would require further evidence.

In practice, our study suggests that teleconsultation has a positive effect on the quality of relationships among doctors with different roles, which should in turn lead to better coordination outcomes. These findings call for a critical organizational re-designing of health services. Managers and health professionals often struggle to implement large-scale, mature teleconsultation services, as they encounter policy, governance, organizational, and cultural hinders. However, one of the pivotal issues in designing health services that embed digital technologies is to understand the mechanisms through which they can be significantly beneficial for their users. In the case of doctor-to-doctor teleconsultation, these mechanisms substantially consist of higher-quality communication, which in turn feeds high-quality relationships that are based on shared knowledge of each other’s role, shared understanding of the overall care process and mutual respect of each other’s contribution to the process. Coherently with the relational coordination approach, we encourage healthcare organizations planning to introduce teleconsultation (and, more generally, digital technologies) within coordinated care processes to consider the impact of its introduction on the relationships among the roles that are involved and to measure it at every relevant point in time.

Finally, although it has been proved that teleconsultation can be beneficial in many ways, it is also true that these tools are not quite diffused. Within our sample of respondents, not even half of the specialist doctors used teleconsultation and only a small percentage declared to do it regularly. Three ‘infrastructural’ actions are required in this sense: (i) the large-scale provision of appropriate digital infrastructures and software to conduct teleconsultations; (ii) the targeted development of enabling competencies for the use of these tools, through the embodiment of digital skills development courses within university curricula and on-the-job training; (iii) the design of appropriate incentive systems for the use of teleconsultation, including reimbursement schemes.

## Conclusions

This study contributes to the ongoing debate on RCT by adding evidence on the cross-cutting structures that affect relational coordination [19], particularly within the current debate on the effect of digitally mediated coordination mechanisms on relational coordination [8]. Moreover, our insights are practically relevant for decision-making concerning coordination processes in the healthcare context, to bridge the gaps associated to the growing specialization of medical expertise as well as the emergent complexity of patient needs. Digital technologies should be exploited as assets to review existing processes in order to make them sustainable.

We are aware of the main limitations of this research. First, the sample selection relied on a large database of contacts that yielded a reasonable number of responses but a low response rate. Second, the decision not to choose single (or multiple) closed sites for survey administration required the formulation of a scenario – within the survey – that had to be detailed enough to avoid the risk of inconsistent responses. Third, as we relied only on survey data, we do not dispose of the actual number of teleconsultations performed by the respondents and we must rely on their perception regarding the frequency of use.

Future studies following the direction of this research could gather the viewpoint of other professionals (e.g., GPs and nurses), as our research concentrated on the one of specialist doctors. Moreover, it should be noted that our model was intentionally designed to be parsimonious. However, future works might include additional dimensions that could be relevant for understanding the effect of teleconsultation use on relational coordination, such as the perception of ‘trust’ towards other organizational roles or the technology itself.

On a conclusive remark, we address the idea that teleconsultation may not simply be a ‘medium’, but in many cases an ‘enabler’ of interaction. Historically, healthcare organizations have been departmentalized, due to professional dynamics [34, 35, 61], and ‘loosely coupled’, due to peculiar relationships between governments and peripheral administrations [62]. Therefore, the positive effect of teleconsultation on relational coordination emerges from its capacity to enhance the frequency and quality interactions among professionals who participate in coordination processes but work in different physical locations.

### Supplementary Information

**Supplementary Material 1. **

## Data Availability

The datasets generated and analyzed during the current study are not publicly available. Although we have removed identifying information, we cannot risk identification by making the data available for public inspection, as we guaranteed anonymity to respondents. Datasets could be available from the corresponding author on reasonable request.

## Abbreviations

GP: General Practitioner
RCT: Relational Coordination Theory

## Footnotes

1 Although our survey was not site-specific, we have accounted for the variety of organizational contexts where respondents work in our analysis. Specifically, we incorporated control variables to reflect the type of organization where each doctor is employed. These include binary variables for different types of public and private healthcare organizations, such as local health authorities, hospitals, research hospitals, other public organizations (e.g., social security institutions) and other private organizations (e.g., insurance companies). We tested our model while controlling for these factors, but our findings remained consistent even when these additional variables were included in the analysis. Therefore, we made the decision to maintain a more parsimonious model in our presentation of the results.
