# Supplementary material for: Digitally-mediated coordination in healthcare: the effects of teleconsultation on doctor-to-doctor relational coordination
Source: BMC Health Serv Res. 2024 Feb 28;24:258. doi: 10.1186/s12913-024-10726-5 (PMC10900703; doi:10.1186/s12913-024-10726-5)
Supplement: Supplementary file 1 — Supplementary Material 1. [file 12913_2024_10726_MOESM1_ESM.docx]

***Additional file 1***

**Detail of Items Investigated Through the Survey**

***Demographics and personal information***

1. Age
2. Gender
3. In what type of organization do you work?

- Local health authority
- Hospital
- Research hospital
- Individual/private practice
- Other public organizations (e.g., social security institutions):
- Other private organizations (e.g., insurance companies):

***Relational Coordination***

*In your daily practice, you likely encounter scenarios in which chronic patients are managed in different settings—such as outpatient specialty clinics and general medicine offices. The statements below aim to capture various aspects concerning your perception of interactions with your colleagues in these scenarios. Please think of a recent, specific case where you had a shared responsibility for a patient who was treated in a multidisciplinary way in different settings (e.g., within integrated care pathways). Using that case as a reference, indicate your level of agreement with each of the following statements about coordination with general practitioners and other specialists.*

1. **How *frequently* did you communicate with each of these care providers about this patient?**

| **General Practitioner** | Never | Rarely | Occasionally | Often | Constantly |
| --- | --- | --- | --- | --- | --- |
| **Other Specialist Doctors** | Never | Rarely | Occasionally | Often | Constantly |

1. **Did these care providers communicate with you in a *timely* way about this patient?**

| **General Practitioner** | Never | Rarely | Occasionally | Often | Constantly |
| --- | --- | --- | --- | --- | --- |
| **Other Specialist Doctors** | Never | Rarely | Occasionally | Often | Constantly |

1. **Did these care providers communicate with you *accurately* about this patient?**

| **General Practitioner** | Never | Rarely | Occasionally | Often | Constantly |
| --- | --- | --- | --- | --- | --- |
| **Other Specialist Doctors** | Never | Rarely | Occasionally | Often | Constantly |

1. **When issues arose regarding the care of this patient did these care providers work with you to *solve the problem*?**

| **General Practitioner** | Never | Rarely | Occasionally | Often | Constantly |
| --- | --- | --- | --- | --- | --- |
| **Other Specialist Doctors** | Never | Rarely | Occasionally | Often | Constantly |

1. **How much did these care providers *know* about your role in caring for this patient?**

| **General Practitioner** | Nothing | Little | Some | A lot | Everything |
| --- | --- | --- | --- | --- | --- |
| **Other Specialist Doctors** | Nothing | Little | Some | A lot | Everything |

1. **How much did these care providers *respect* your role in caring for this patient?**

| **General Practitioner** | Not at all | A little | Somewhat | A lot | Completely |
| --- | --- | --- | --- | --- | --- |
| **Other Specialist Doctors** | Nothing | Little | Some | A lot | Everything |

1. **How much did these care providers *share your goals* for the care of this patient?**

| **General Practitioner** | Not at all | A little | Somewhat | A lot | Completely |
| --- | --- | --- | --- | --- | --- |
| **Other Specialist Doctors** | Nothing | Little | Some | A lot | Everything |

***Frequency of use of teleconsultation***

1. **How often did you recur to teleconsultation in the last year with these care providers in the last year?**

Please refer to teleconsultation in the case of remote interaction through digital tools (phone calls are not to be considered) with other physicians to share medical decisions regarding a patient.

| **General Practitioner** | Never | Rarely (A few times a month) | Occasionally (A few times a week) | Regularly (On a daily basis) |
| --- | --- | --- | --- | --- |
| **Other Specialist Doctors** | Never | Rarely (A few times a month) | Occasionally (A few times a week) | Regularly (On a daily basis) |
